# Comparison of Dydrogesterone and GnRH Antagonists for Prevention of Premature LH Surge in IVF/ICSI Cycles: A Randomized Controlled Trial 

**Published:** 2020-03

**Authors:** Batool Hossein Rashidi, Azam Tarafdari, Seyedeh Tahereh Ghazimirsaeed, Ensieh Shahrokh Tehraninezhad, Fatemeh Keikha, Bita Eslami, Seyede Masomeh Ghazimirsaeed, Mina Jafarabadi

**Affiliations:** 1 Reproductive Health Research Center, Tehran University of Medical Sciences, Tehran, Iran; 2 Breast Disease Research Center, Tehran University of Medical Sciences, Tehran, Iran; 3 Shahid Aliabadi Clinical Research Center, Iran University of Medical Sciences, Tehran, Iran

**Keywords:** Controlled Ovarian Stimulation, Dydrogesterone, Gonadotropin Releasing Hormon Antagonist, Premature luteinizing Hormone Surge

## Abstract

**Objective:** To compare the effect of dydrogesterone and Gonadotropin releasing hormone (GnRH) antagonists on prevention of premature luteinizing hormone (LH) surge and pregnancy outcomes in infertile women undergoing Invitro fertilization/ Intra cytoplasmic sperm injection (IVF/ICSI).

**Materials and methods:** In a Randomized controlled trial (RCT), two-hundred eligible women undergoing in vitro fertilization (IVF) /intracytoplasmic sperm injection (ICSI) treatment were randomly assigned into two groups. Human menopausal gonadotropin (HMG) was administered for controlled ovarian stimulation (COS) in both groups. Intervention group (group 1) received 20 mg dydrogesterone from day 2 of menstrual cycle till trigger day and control group (group2) received GnRH antagonist from the day that leading follicle reached 13 mm in diameter till trigger day. Serum levels of LH, estradiol and progesterone were measured on the trigger day. The primary outcome measure was the incidence of a premature LH surge, and the secondary outcomes investigated were the chemical and clinical pregnancy rates in the first FET cycles.

**Results:** There were no significant differences in patients' age, BMI, AMH levels, previous IVF cycle, and cause of infertility between the two groups. None of the patients in two groups experienced a premature luteinizing hormone surge. The numbers of retrieved oocytes, the MII oocytes and good quality embryos, were significantly higher in the intervention group than antagonist group (p < 0.05). The overall chemical pregnancy rate in intervention group (43/91: 46.2%) and control group (45/91: 49.5%) (p = 0.820) was similar. Meanwhile, the clinical pregnancy rate was similar between groups too.

**Conclusion:** Regarding the cost, efficacy and easy usage of dydrogestrone, it may be reasonable to use it as an alternative to GnRH antagonist for the prevention of premature LH surge.

## Introduction

During the last few decades, assisted reproductive technology (ART) has been widely used in infertile women. Controlled Ovarian stimulation (COS) is one of the most important challenges to induce adequate number of retrieved oocytes in order to increase the efficacy of ART (1, 2).

During controlled ovarian stimulation (COS), suppressing the premature luteinizing hormone (LH) surge which is responsible for 20-50% of cycle cancellation, could improve the ART outcomes (3, 4). Gonadotropin-releasing hormone (GnRH) analogue protocols are commonly used to prevent premature LH surge and decrease the cycle cancellation rate (5, 6). 

However pituitary gland desensitization by GnRH agonist has several disadvantages including long-term and higher doses of gonadotropines, side effects resulting of estrogen deficiency, and cost consuming (7, 8). GnRH antagonist that recently introduced for LH surge suppression in ART cycles, is more effective and can prevent ovarian hyperstimulation syndrome (OHSS) (9, 10). This protocol has fewer complications, and lower number ofGnRH injections which is more convenient for patients. However, it is expensive and occasionally premature LH surge, undesired ovulation, and cycle cancellation were reported in some patients (11, 12). So, its effectiveness is still under debate (13).

Therefore, new regimens with improved efficacy and patients' convenience are needed to be established to prevent premature LH surges and research is still ongoing (13).

Previous studies have reported the preventive effect of several types of progestins on premature LH secretion in controlled ovulation stimulation cycles (14-17). Progesterone accompanied to estrogen has a role in suppression preovulatory LH surge. However, the timing of progesterone administration is critical in determining its effect upon the pre-ovulatory LH surge, whether it is stimulating or inhibiting (13). In previous years, progesterone was not used during ovarian stimulation due to negative effect on endometrial receptivity, while newly advanced verification techniques have suggested it as an alternative to a GnRH analogue for suppressing premature LH surges during ovarian stimulation in in vitro fertilization (IVF) cycles using the freeze-all strategy and transfer of thawing cryopreserved embryo (13).

Dydrogesterone as a synthetic progestin that is closely related to endogenous progesterone is a more patient-friendly regimen with some advantages such as: oral administration, less androgenic activity, fewer adverse effects compared to other progestins, and highly selective for the progesterone receptor (18-20). Only a few studies have demonstrated the efficacy of a combination of dydrogesterone and human menopausal gonadotropin (HMG) for progestin-primed ovarian stimulation (PPOS) in IVF cycles (16, 21).

Therefore, this study was conducted to compare the effect of oral dydrogesterone plus HMG (intervention group) with a standard GnRH antagonist protocol (control group) in prevention of premature LH surge and pregnancy rates in infertile Iranian women.

## Materials and methods

***Study setting and patients' allocation: ***The Ethics Committee of Tehran University of Medical Sciences approved this study and appropriate informed consent was obtained from all participants. This randomized controlled trial (RCT) has been registered with the Iranian Registry of Clinical Trials (Registration ID. IRCT20181031041519N2).

Two-hundred infertile women who were candidate for IVF/ ICSI cycles at a university based infertility center between September 2018 to October 2019, were recruited in this study. The inclusion criteria were as follows: age younger than 40 years, anti-mullerian hormone (AMH) levels greater 1.5 ng/ml, body mass index (BMI) less than 30 kg/m^2^, presence of both ovaries, and antral follicle count (AFC) more than 5-7 on day 1–3 of the menstrual cycle.

The exclusion criteria were: smoking, patients with uterine anomalies, adrenal insufficiency, uncontrolled thyroid disease, endometriosis stage ≥ 3, hyperprolactinemia, neoplastic ovarian disorders, repeated implantation failure (RIF), and severe male factor infertility.

The eligible women were allocated consequently to one of the two groups: intervention or progestin primed ovarian stimulation (PPOS) group (dydrogesterone + hMG; n = 100) and control group (GnRH antagonists + hMG; n = 100).

***Study clinical protocol: ***The two study groups were well defined and randomly assigned. The intervention group subjects received dydrogesterone (Duphaston; Abbot Co, Netherlands) 20 mg daily and HMG (Merional, IBSA Co., Switzerland) 150-225 units every 24 hours from day 2-3 of menstrual cycles, till the trigger day. (Trigger day is a day in IVF cycles when egg development takes place during gonadotropin stimulation then a trigger shot is given about 36 hours prior to egg retrieval).

The control group subjects also received HMG (Merional, IBSA Co. Switzerland) 150-225 units from day 2-3 menstrual cycle and when the leading follicle reached 13 mm, GnRH antagonist (Cetrotide; Mreck, Serono, Germany) 0.25 mg was injected subcutaneously every 24 hours, till the trigger day.

In both groups patients were monitored by every other day transvaginal sonography starting on day 5 of stimulation. Infertility clinician, who was blind to the allocation of groups, performed the monitoring.

On the trigger day when sonographic monitoring showed the minimum of two 18 mm follicles, a blood sample was taken to check the LH, estradiol and Progesterone serum levels and then Triptorelin (Decapeptyl; Ferring, Germany) 0.2 mg was injected subcutaneously.

Oocyte pick up was performed from posterior vaginal fornix 34 to 36 hours after triptorelin administration. Then, the retrieved oocytes were classified by an embryologist according to maturation of oocytes. Two to three days after ICSI, the embryos were scored based on the cell number and fragmentation by the embryologist. Good quality embryos were frozen and transferred in the next cycle. 

All patients underwent FET cycles and hormone replacement therapy was performed for endometrial preparation. Estradiol valerate (Aburaihan Co. Iran) 6 mg daily was started from day two of the menstrual cycles and was increased to 8 mg daily if endometrial thickness did not reach at least 8 mm in transvaginal sonography.

Whenever the endometrial thickness was more than 8 mm progesterone suppository (Cycogest; Actavis, England, UK) 400mg twice daily was started and continued till 10 weeks of gestation. Two good quality cleaved embryos were transferred for all participants and all embryo transfers were performed by an expert infertility clinician. 

Two weeks after embryo transfer, serum B-HCG was assessed by the same laboratory kit. It should be noted that the LH and progesterone levels were measured on the trigger day and chemical pregnancy was also investigated in the first cycle of embryo transfer. Post trigger LH concentration at or near 15µ/mL is used as a threshold for LH surge assessment (22, 23). The criteria for cycle cancelation were no mature follicles for oocyte pick up.

The primary outcome was the suppression of premature LH surge and the secondary outcomes were the chemical (positive of pregnancy test) and clinical pregnancy rate (viable intrauterine pregnancy till 12 weeks of pregnancy). The early abortion rate was defined as the proportion of pregnancies arresting before 12 weeks of gestation. 

***Statistical analysis: ***Statistical analysis was performed using SPSS software 24 (Version 24, SPSS, Inc, IL, USA). In descriptive analysis we used, mean ± standard deviation (SD) for quantitative and number (%) for qualitative variables. In analytical analysis, independent samples t-test or Mann-Whitney U-test was conducted considering the normality of data and Chi-squared test was used for qualitative variables. P-value of < 0.05 was considered statistically significant.

## Results

**Figure 1:**
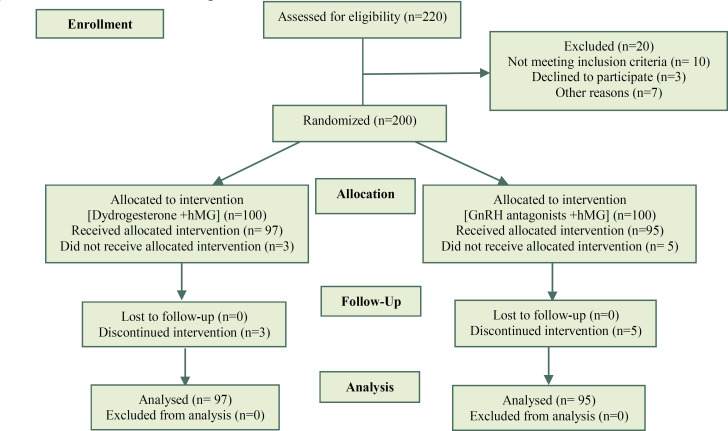
CONSORT flowchart of patients enrolled in the study

Figure 1 shows the CONSORT flowchart of the women enrolled in the study. Three patients in intervention group and five patients in control group didn't develop follicle due to the lack of drug consumption and excluded from the study analysis. Therefore, final analysis was conducted in 97 and 95 patients in intervention and control group, respectively. Lack of oocytes (no oocyte) in ovum pick up (OPU) and embryo development (no embryo) was observed in six and four patients in intervention and control group, respectively. There were no cases of premature LH surge and OHSS in both groups. 

There were no significant differences in patients' age, BMI, AMH, previous IVF cycle, cause of infertility between two groups (Table 1). The numbers of retrieved oocytes, metaphase II oocytes (MII) and the good quality embryos were significantly higher in the intervention group compared with control group (p < 0.05). The mean values of serum estradiol, LH, and progesterone on the trigger day were significantly higher in the intervention group compared to the control group (p < 0.05). No statistically significant differences were observed in fertilization rates (which mean transformation of micro injected oocytes into two pronuclei), chemical pregnancy, clinical pregnancy, and abortion rate between two groups (Table 2). 

## Discussion

The present study results manifested that COS with dydrogesterone was statistically similar to the GnRH antagonist protocol regarding to the primary (premature LH surge) and secondary outcomes (pregnancy rate).

Our results were similar to Iwami et al. study in design and results of the study. They also showed no significant differences in ongoing pregnancy rate (40 vs.38.1%) and clinical pregnancy rate (52.8 vs. 49.5%) between two treatment regimens. Moreover, our results about premature LH surge was in line with the above (5). 

**Table 1:** Baseline and obstetric variables in intervention and control groups

**Variables **	**Intervention ** **(n= 97)**	**Control ** **(n=95)**	**P-** **value**
Age (Year)	32.71 ± 4.63	33.20 ± 5.21	0.279
BMI (Kg/m^2^)	24.19 ± 2.92	25.53 ± 3.32	0.580
AMH (ng/ml)	4.70 ± 3.81	3.97 ± 2.70	0.174
Duration of Infertility (Year)	5.15 ± 3.20	5.55 ± 2.97	0.248
Previous IUI cycles	1.77 ± 0.54	1.80 ± 0.79	0.915
Previous IVF cycles	0.67 ± 0.73	1.00 ± 0.99	0.188
Previous cycles of embryo transfer	0.61 ± 0.71	0.95 ± 0.85	0.138
Type of Infertility			
Primary	74 (76.28)	82 (86.31)	0.034
Secondary	23 (23.71)	13 (13.68)	
Cause of infertility			
Male	24(24.74)	22 (23.15)	0.032
Female	34 (35.05)	37 (40.65)	
Both	16 (16.49)	14 (14.73)	
Unknown	23 (23.71)	22 (23.15)	
Cause of female Infertility			
TFI	10 (29.5)	10 (27)	0.123
PCO	17 (50.0)	23 (62.2)	
Other	7 (20.5)	4 (10.8)	

Data presented as mean ± SD and number with percentages in parenthesis when appropriate. Independent T test and Chi square test were used.

TFI: Tubal factor infertility; PCO: Polycystic ovary syndrome

However, another study by Huang et al. had contradictory results with our findings. They conducted a retrospective study that compared the method of PPOS with a GnRH antagonist in poor responders undergoing in IVF/ICSI. They concluded that the PPOS protocol can effectively improve clinical pregnancy and live birth rates compared with the GnRH antagonist protocol (24). It should be considered that their results may not be applicable in all patients because they selected only the poor responders. 

Some studies have investigated different types of progesterone in order to LH suppression during ovarian stimulation. Yu et al. in a prospective study evaluated the effect of dydrogesterone and medroxyprogesterone acetate (MPA) in PPOS protocol in 516 first IVF/ISCI cycles. No patients experience premature LH surge and moderate or severe OHSS. Their findings suggested no significant differences between two group outcomes and they concluded dydrogesterone could use as an alternative progestin for PPOS protocol in IVF (21). Unlike this study, Beguería et al. has reported the lower reproductive outcomes with MPA compared with a GnRH antagonist (25, 26). This difference may be related to the type, dose and the timing of administration of progestin.

**Table 2:** Comparison of outcomes in intervention and control groups

**Variables **	**Intervention group (n= 97)**	**Control group (n=95)**	**P-value**
	**Mean ± SD**	**Mean ± SD**	
Number of HMG vials in cycle	34.68 ± 8.13	32.03 ± 7.11	0.003
Number of HMG injection days	11.90 ± 1.90	11.34 ± 1.72	0.013
Number of retrieved oocytes	9.45 ± 4.12	7.26 ± 4.02	< 0.001
Number of metaphase II oocyte	7.90 ± 3.62	6.26 ± 3.64	< 0.001
Number of embryos	6.54 ± 2.77	5.32 ± 3.09	0.001
Fertilization rate (Number of 2 pronuclei/number of metaphase II oocyte)	0.816	0.849	0.650
Number of Grade A embryos	3.19 ± 1.70	3.28 ± 1.88	0.945
Number of quality embryos in Grade B	2.14 ± 0.81	1.90 ± 0.90	0.211
Number of quality embryos in Grade AB	2.50 ± 0.91	2.36 ± 0.80	0.296
Estradiol level on trigger day (pg/ml)	3002.84 ± 1933.11	2171.91 ± 2103.43	< 0.001
LH on trigger day (mIU/ ml)	1.82 ± 1.40	1.10 ± 0.60	< 0.001
Progesterone on trigger day (ng/mL)	1.42 ± 0.70	0.78 ± 0.28	< 0.001
	**N (%)**	**N (%)**	
Chemical pregnancy in first embryo transfer	43 (46.20)	45 (49.50)	0.820
Clinical pregnancy in first embryo transfer	40 (43.95)	41 (45.05)	0.881
Abortion frequency	3 (3.29)	4 (4.39)	0.700
Number of Cancellation of cycle	3(3.29 )	5(5.49)	0.470
No oocyte (lack of oocyte in ovum pick up)	2(2.19 )	2(2.19 )	0.990
No embryo (no embryo development after intracytoplasmic sperm injection)	4 (4.39)	2(2.19 )	0.406

Manwithney test and Chi-Square test were used. SD: Standard deviation

Zhu et al. conducted a study for COH by two regimens: oral dydrogestrone + hMG (intervention group) and Utrogestan + hMG (control group) during IVF/ICSI. This study results found dydrogestrone is similar to Utrogetan in prevention of LH surge, embryonic characteristics, and pregnancy outcomes (16).

Finally, in 2019 La Marca and Cauzzo reviewed studies which reported the use of exogenous progestins in ovarian stimulation. They concluded reproductive outcomes from ovarian stimulation with progestins are similar to those from conventional ovarian stimulation, although they thought large trials are needed to confirm this. It seems progestins can suppress a premature LH-surge during follicular phase with lower cost, safe and easier administration (oral), and similar effectiveness and can be used as an alternative to GnRH analog. Despite the advantages of PPOS, it has some weaknesses such as a delayed embryo transfer and higher dose of gonadotropins used. They suggested further studies especially on neonatal outcomes are needed before this protocol can be introduced on a wider scale (13).

## Conclusion

The present study revealed an equal effect of using 20 mg/day of dydrogestrone and GnRH antagonist on prevention of premature LH-surge and embryonic and reproductive outcomes. Therefore, it seems dydrogesterone is a reasonable option for ovarian stimulation. However, more clinical trials are necessary to determine the optimal dose and the best time for starting dydrogestrone in ovarian stimulation cycles, in normal and poor responders as well as elder patients. 
